# TRAIL induces apoptosis in oral squamous carcinoma cells: a crosstalk with oncogenic Ras regulated cell surface expression of death receptor 5

**DOI:** 10.18632/oncotarget.813

**Published:** 2013-02-23

**Authors:** Jun-jie Chen, Constantinos M. Mikelis, Yaqin Zhang, J. Silvio Gutkind, Baolin Zhang

**Affiliations:** ^1^ Division of Therapeutic Proteins, Office of Biotechnology Products, Center for Drug Evaluation and Research, Food and Drug Administration, Bethesda, MD; ^2^ Oral and Pharyngeal Cancer Branch, National Institute of Health, Bethesda, MD

**Keywords:** oral cancer, TRAIL, death receptors, apoptosis, Ras, oncotarget

## Abstract

TNF-related apoptosis inducing ligand (TRAIL) induces apoptosis through its death receptors (DRs) 4 and/or 5 expressed on the surface of target cells. The selectivity of TRAIL towards cancer cells has promoted clinical evaluation of recombinant human TRAIL (rhTRAIL) and its agonistic antibodies in treating several major human cancers including colon and non-Hodgkin's lymphoma. However, little is known about their ability in killing oral squamous cell carcinoma (OSCC) cells. In this study, we tested the apoptotic responses of a panel of seven human OSCC cell lines (HN31, HN30, HN12, HN6, HN4, Cal27, and OSCC3) to rhTRAIL and monoclonal antibodies against DR4 or DR5. We found that rhTRAIL is a potent inducer of apoptosis in most of the oral cancer cell lines tested both *in vitro* and *in vivo*. We also showed that DR5 was expressed on the surface of the tested cell lines which correlated with the cellular susceptibility to apoptosis induced by rhTRAIL and anti-DR5 antibody. By contrast, little or no DR4 was detected on the surface of OSCC3 and HN6 cells rendering cellular resistance to DR4 antibody and a reduced sensitivity to rhTRAIL. Notably, the overall TRAIL sensitivity correlated well with the levels of endogenous active Ras in the cell lines tested. Expression of a constitutively active Ras mutant (RasV12) in OSCC3 cells selectively upregulated surface expression of DR5, but not DR4, and restored TRAIL sensitivity. Our findings could have implications for the use of TRAIL receptor targeted therapies in the treatment of human OSCC tumors particularly the ones harboring constitutively active Ras mutant.

## INTRODUCTION

Oral squamous cell carcinoma (OSCC) is the most common oral and pharyngeal cancer that affects approximately 500,000 new cases annually worldwide [1, 2]. Despite the aggressive management including surgery, radiation, and chemotherapy [3], most patients with advanced OSCC develop local or regional recurrences and distant metastasis that accounts for a poor prognosis that has remained unchanged for decades [4, 5]. There is an unmet need for more effective and less toxic molecularly targeted therapies for treating this disease.

TNF-related apoptosis inducing ligand (TRAIL) induces apoptosis through its death receptors (DRs) 4 and/ 5 expressed on the surface of target cells. Both receptors are characterized by a cytoplasmic death domain that, upon ligand binding, facilitates the assembly of the death-inducing signaling complex (DISC) involving procaspase-8 or -10 [6, 7]. Within the DISC, procaspase-8 or -10 is self-cleaved and activated, resulting in subsequent activation of a caspase cascade, cleavage of cellular proteins, and eventually apoptotic cell death. Preclinical data indicate that recombinant human TRAIL (rhTRAIL) can induce apoptosis in a broad range of human cancer cell lines while sparing most normal cell types [8-11]. In tumor xenograft models, rhTRAIL exhibits single-agent antitumor activity and/or cooperation with certain conventional and targeted therapies [11-14]. These unique properties of TRAIL have promoted multiple clinical trials that are currently ongoing to test rhTRAIL and monoclonal antibodies to DR4 or DR5 for the treatment of various malignancies, including melanoma, ovarian, renal, and colorectal cancers [15, 16]. However, several issues remain to be resolved such as the identification of tumor types that are mostly susceptible to TRAIL based therapies, and the detection of biomarkers predictive of tumor sensitivity. Indeed, little is known about the apoptosis-inducing activity of rhTRAIL and its agonistic antibodies in OSCC cells.

The utility of TRAIL as a cancer therapy is limited by the development of drug resistance in cancer cells. Many factors have been reported to affect TRAIL-induced apoptosis, including decoy receptors DcR1, DcR2 and OPG that lack a death domain but compete with DR4/DR5 for TRAIL binding, the expression of inhibitory proteins in signal transduction pathways such as c-FLICE-inhibitory protein (FLIP) [17] and IAP family proteins [18], and deficiency in caspase-8 [19]. We and others have recently shown that the post-translational modifications of DR4 and DR5 receptors, including O-glycosylation [20] and endocytosis [21-23], are also a critical determinant of cellular sensitivity to TRAIL based therapies. Recent evidence suggests that oncogenic Ras may also be involved in the regulation of TRAIL receptors, thereby sensitizing cancer cells to TRAIL-induced apoptosis [24-26]. Ras signaling is often activated in human tumors including OSCC [27-29], where it contributes to the tumorigenicity through interaction with various downstream effector targets, such as MEK, PI3K, and Rho GTPases [30, 31]. Ras regulates a Raf-MEK-ERK1/2 kinase cascade which has been shown to sensitize colon cancer cells to TRAIL-induced apoptosis by up-regulating DR4 and DR5 [24, 25]. TRAIL resistance is also seen in OSCC cell lines [32-35], and the underlying mechanisms remain to be better understood.

To explore the potential of targeting death receptors against OSCC, we tested the apoptosis-inducing activity of rhTRAIL and its agonistic antibodies in a panel of OSCC cell lines. We found a positive association between TRAIL sensitivity, surface expression of DR4 and DR5, and the levels of activated Ras, with the TRAIL-resistant cell lines OSCC3 being found to be of the lowest levels of surface DR4/DR5 and Ras activity. Interestingly, transfection of OSCC3 cells with a constitutively Ras mutant specifically increased the surface expression of DR5, but not DR4, and sensitized the cells to DR5 monoclonal antibody and rhTRAIL-induced apoptosis. The results suggest that rhTRAIL and its agonistic antibodies could be used in cases of human OSCC tumors bearing activated Ras pathways.

## RESULTS

### TRAIL is a potent inducer of apoptosis in some oral cancer cell lines

**Figure 1:**
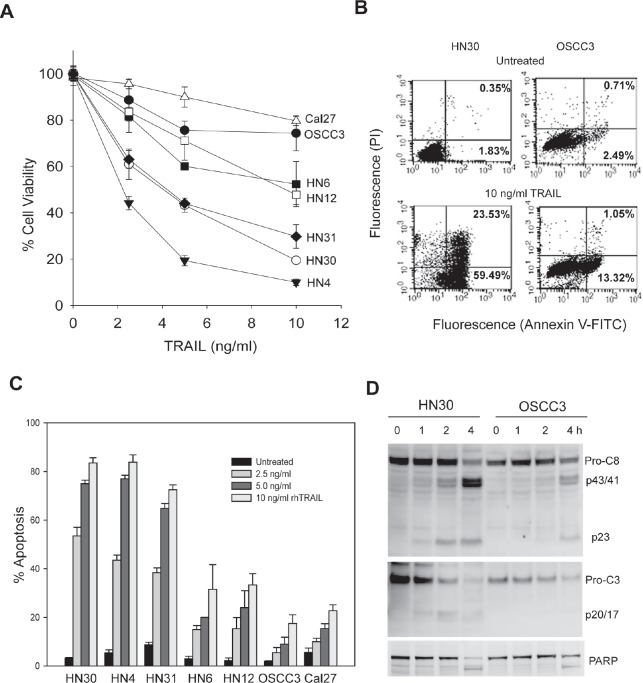
TRAIL-induced cytotoxicity in oral cancer cell lines A, Cells were treated with increasing doses of TRAIL for 6 h, followed by MTT assay, and cell viability was reported as percentages of corresponding untreated cell lines. B, Flow cytometry analysis of apoptosis in HN30 and OSCC3 cells untreated or treated with 10 ng/ml TRAIL for 6 h. *Bottom right quadrant*, percentage of early apoptotic cells with exposed phosphatidylserine (Annexin V-FITC positive) but intact membrane (propidium iodide negative). *Top right quadrant*, necrotic or apoptotic cells in terminal stages with positive staining of both Annexin V-FITC and propidium iodide. C, Quantification of TRAIL-induced apoptosis (as described in B) in the indicated cell lines. Mean ± SD total percentage of the cells in the right quadrants (n=3). D, Differential activation of caspase-8 and -3 in HN30 and OSCC3 cells treated with 10 ng/ml TRAIL for the indicated times. Caspase activity is indicated by the decrease of pro-caspases (pro-C8 and pro-C3) and appearance of the p23 and the p20/p17 cleaved forms of caspase-8 (C-8) and caspase-3 (C-3), as well as the cleavage of poly (ADP-ribose) polymerase (PARP), a caspase-3 substrate.

To assess the ability of TRAIL in killing OSCC cancer cells, we first determined cell viability in a panel of seven representative human oral cancer cell lines. As shown in Fig. 1A, TRAIL induced a dose-dependent decrease in cell viability in all the cell lines tested although the sensitivity is different. HN4, HN31 and HN30 showed the highest sensitivity to rhTRAIL whereas only minimal cytotoxicity was detected in OSCC3 and Cal27 cells. The difference in TRAIL sensitivity was further supported by flow cytometry assays, demonstrating differential apoptotic responses to rhTRAIL between the cell lines. At a concentration as low as 10 ng/mL of rhTRAIL, HN4, HN31 and HN30 cell lines underwent rapid and substantial apoptosis indicated by Annexin V binding (Fig. 1B and C) and activation of caspase-8 and caspase-3 and cleavage of a caspase substrate PARP (Fig. 1D). By contrast, other cell lines had modest to little apoptosis in response to rhTRAIL treatment. These results indicate that rhTRAIL is a potent inducer of apoptosis in certain OSCC cell lines.

### TRAIL-sensitive cells are susceptible to agonistic antibodies to DR4 and DR5

**Figure 2:**
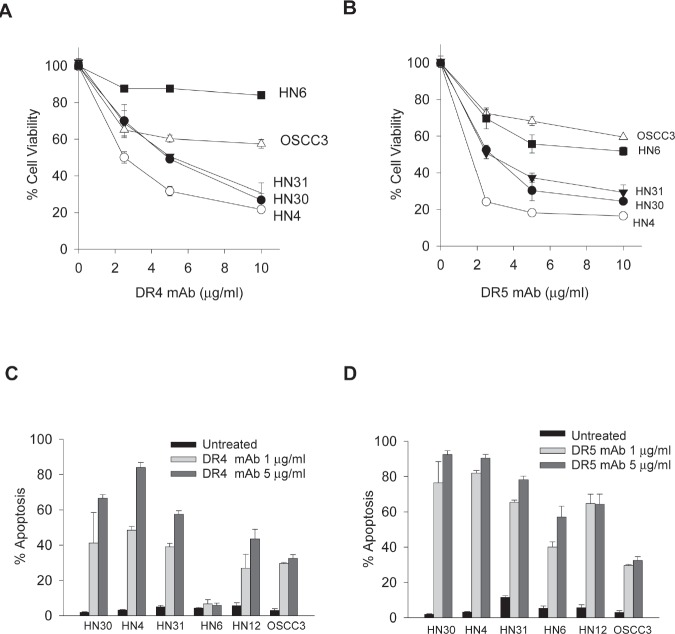
Apoptotic responses to agonistic DR4 and DR5 monoclonal antibodies A & B, Cell viability of the indicated cell lines treated with increasing doses of DR4 monoclonal antibody (DR4 mAb) (A) or DR5 mAb (B) for 24 h. C & D, Apoptosis of the indicated cell lines treated with DR4 mAb (C) or DR5 mAb (D) antibodies at 1 or 5 μg/mL for 24h (Mean ± SD (n=3).

Next, we tested the cellular responses to the agonist antibodies against DR4 or DR5. The results showed that TRAIL-sensitive cell lines (HN31, HN30 and HN4) were selectively sensitive to both DR4 and DR5 monoclonal antibodies (Figs. 2A & 2B), which correlated with the extent of apoptosis (Figs. 2C & 2D) in the corresponding samples. Anti-DR5 antibody also induced growth inhibition and apoptosis in HN6, HN12 and OSCC3 lines (Fig. 2B & 2D). However, anti-DR4 antibody showed a different cytotoxicity profile where it induced apoptosis in all five cell lines, but not HN6 (Fig. 2A & 2C). The resistance of HN6 cells persisted at higher concentrations of anti-DR4 up to 100 μg/mL (data not shown). Nevertheless, these results demonstrate a significant number of OSCC cell lines that are susceptible to both rhTRAIL and its agonistic antibodies. The results warrant additional studies to assess the potential utility of TRAIL receptor targeted biologic agents in the treatment of oral squamous cell carcinomas.

### *In vivo* antitumor activity of TRAIL

**Figure 3:**
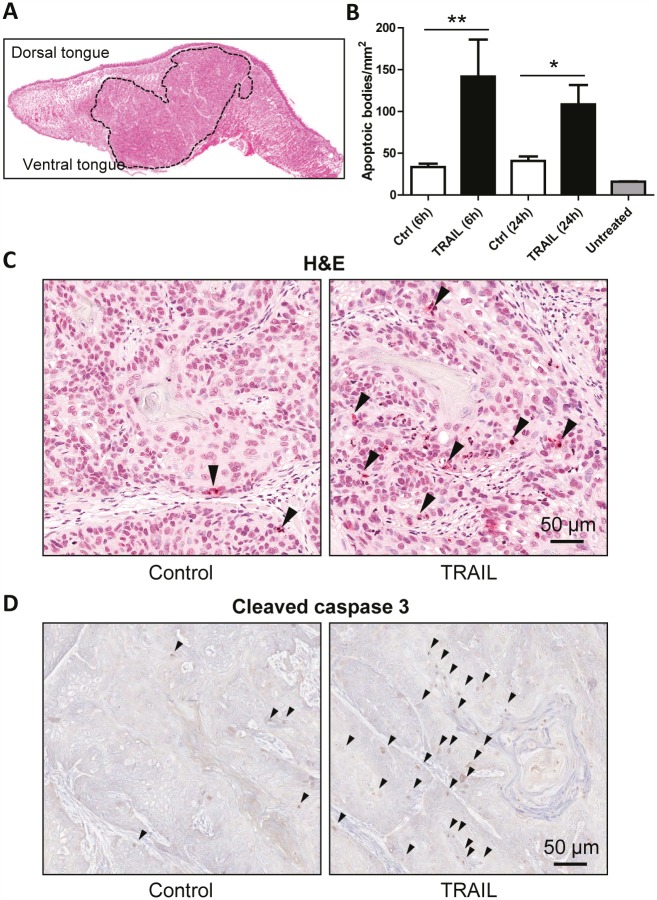
TRAIL induces apoptosis in orthotopic tumor xenografts A, Local invasive tumor growth of orthotopically implanted HN31 oral cancer cells into the tongue. H&E stained tissue section of an oral cancer orthotopic tumor (delimited with dotted line). B, Quantification of TRAIL-induced apoptosis, as assessed by TUNNEL assay in the tumor samples. C, Representative images of paraffin sections stained for apoptosis with TUNNEL assay. D, Representative images of paraffin sections stained for active (cleaved) caspase-3 (Abcam #ab2302).

To determine the *in vivo* activity of rhTRAIL, we established an orthotopic tumor xenograft model of OSCC using HN31 cells as described previously [38]. The cells were injected into the dorsal tongue of SCID/NOD mouse and the tumor lesions were measured weekly up to 5 weeks (Fig. 3A). Animals with similar tumor size (80 mm^3^) in tongue were treated with a vehicle or rhTRAIL. After intratumoral injection, TRAIL induced a time-dependent apoptosis in tumor tissues as indicated by the onset of apoptotic bodies inside the tumor as well as an accumulation of the cleaved and active form of caspase-3, a hallmark of TRAIL-induced apoptosis (Fig. 3B & D). These observations are in line with the *in vitro* data in Fig. 1 demonstrating a potency of rhTRAIL in inducing apoptosis in oral cancer cells.

### Surface DR4/5 expression is a critical determinant of cellular sensitivity to TRAIL receptor targeted therapies

**Figure 4:**
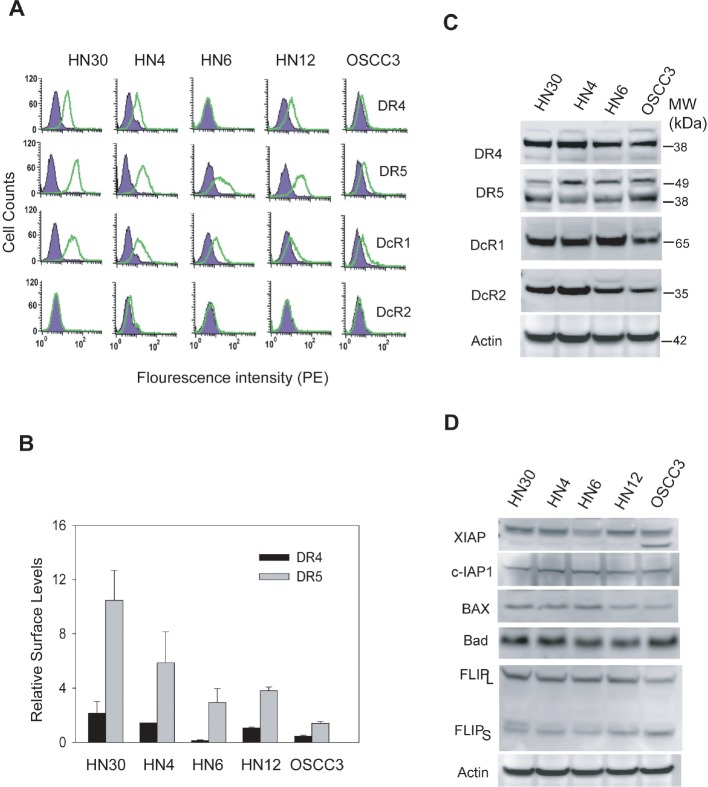
Differential expressions of TRAIL receptors on cell surface A, The expressions of TRAIL receptors on cell surface were determined by flow cytometry analysis using PE-conjugated antibodies specific to DR4, DR5, DcR1 or DcR2 (*open histogram*). The presence of the receptor on cell surface is indicated by a right-shift of the peak compared to cells stained with isotype-matched control IgG-PE (*shadowed histograms*). B, Quantification of results in A reveals the relative levels of surface DR4 and DR5 by comparing the mean values of PE-anti-DR4 or PE-DR5 antibodies and their corresponding PE-IgG controls. C, Immunoblots showing the total protein levels of TRAIL receptors. Actin was shown as a loading control. D, Immunoblots of several relevant TRAIL signaling components: XIAP, c-IAP-1, Bax, Bad and FLIP. Representative blots of three separate experiments.

The proper expression of TRAIL receptors on the surface of target cells is essential for the action of ligand or antibodies. We have previously shown that TRAIL resistance was associated with a deficiency in DR4 and DR5 surface expression in breast [21-23] and rhabdomyosarcoma [19] cell lines. We asked if this was true in OSCC cells and therefore examined the surface expression of TRAIL receptors (DR4, DR5, DcR1 and DcR2) by flow cytometry using PE-conjugated antibodies specific to each receptor (Fig. 4A & 4B). As expected, both DR4 and DR5 were significantly expressed on the surface of TRAIL-sensitive cell lines (HN4 and HN30). Surface DR5 was also detected in other three cell lines at differential levels. Notably, DR4 was not detected on the surface of HN6 cells and only little on OSCC3 cells, despite its total protein expression across the cell lines (Fig. 4C). The lack of DR4 surface expression in HN6 cells was directly correlated with the observed resistance to anti-DR4 antibody and a reduced sensitivity to rhTRAIL. All the cell lines expressed DcR1 and DcR2 at comparable total protein levels, while DcR1, but not DcR2, was also detected on surface of all the cell lines. However, there was no direct relationship between the levels of decoy receptors and the observed TRAIL sensitivity.

We also tested the expression of several regulator proteins that are involved in the regulation of TRAIL-induced apoptosis signaling pathway. These include cFLIP, IAPs, and the Bcl2 family members which are key regulators of caspase activity and have been implicated in TRAIL resistance in some cancer cell lines [17, 18]. Immunoblots showed that each protein was expressed at a similar level in the OSCC cell lines (Fig. 4D), suggesting that they may play a minor role, if any, in the observed TRAIL sensitivity. Collectively, these results are consistent with our previous observations in other cancer types [19, 21-23] that deficiency of TRAIL death receptors on cell surface is also a critical determinant of TRAIL resistance in OSCC cells.

### Oncogenic Ras activity is associated with TRAIL sensitivity

As with many other cancer types, the development of oral cancer is often accompanied by an upregulated Ras signaling pathway due to gain of function mutations in *ras* genes itself and/or alterations in the regulatory proteins [2, 27-29]. Recent evidence suggests a link between Ras activity and the death receptor mediated apoptosis. For example, transformation by Ras enhanced TRAIL-induced apoptosis in colon cancer cell lines [24, 39]. To explore this possibility in OSCC cells, we first determined the level of endogenous active Ras-GTP by affinity purification using GST-Raf1 immobilized on agarose beads. Notably, the levels of Ras-GTP correlated well with the observed TRAIL sensitivity (Fig. 4A & Table 1). While the total Ras protein remained the same in all the cell lines, Ras-GTP is significantly higher in the TRAIL-sensitive cell lines (HN30 and HN4) than insensitive cell lines.

**Table 1:** Relationship between Ras activity, cell surface expression of DR4 and DR5, and cellular sensitivity to rhTRAIL and agnostic antibodies to DR4 (DR4 mAb) or DR5 (DR5 mAb) in oral cancer cell lines

Cell lines	Surface Levels*		Ras mutant statusφ	Sensitivityξ		
	DR4	DR5		TRAIL	DR4 mAb	DR5 mAb
HN30	+++++	+++++	G12D (K-Ras)	+++++	++++	+++++
HN31	+++	+++++	G12D (K-Ras)	+++++	+++++	+++++
HN4	+++	+++++	G12D (H-Ras)	+++++	++++	+++++
HN6	null	+++	WT	+++	−	++++
HN12	+++	+++	WT	+++	+++	++++
OSCC3	+	+	WT	+	+	+

* Surface expression relative to HN30 cells using the data shown in Fig. 4A and not shown for HN31. Scale from high (+++++) to low (+).

φ H-Ras mutant status was determined by cDNA sequence analysis at Dr. Gutkind laboratory. WT, wild type.

ξ Sensitivity relative to HN30 cells using data in Fig. 1A. -, resistant.

**Figure 5:**
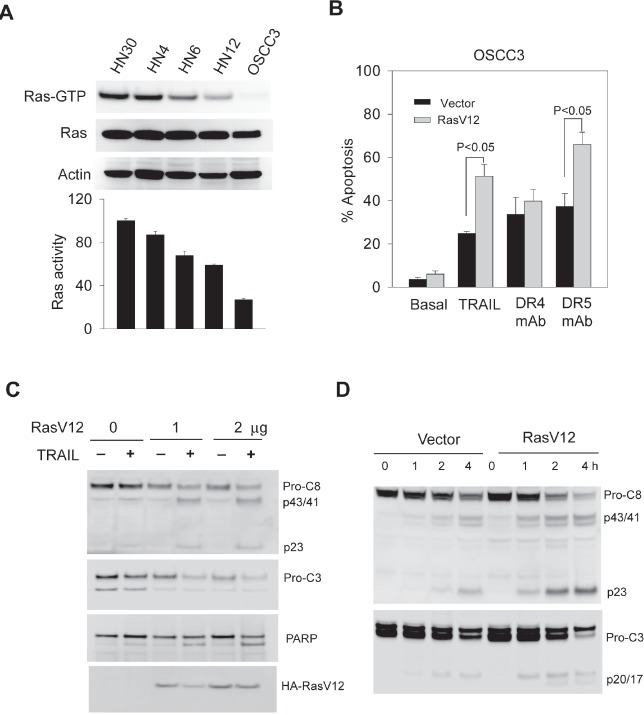
TRAIL sensitivity is associated with Ras activity in the target cells A, The levels of endogenous activated Ras (Ras-GTP) in the indicated cell lines were examined by the GST-Raf pull-down assay using GST-Raf-1 binding domain immobilized on agarose beads (*see* details in Materials and Methods). Affinity precipitates were analyzed by western blotting with an antibody specific to Ha-Ras. *Lower panel*, densitometry analysis of the immunoblots reveals semi-quantitative levels of Ras-GTP and expressed relative to HN30 cells that showed the highest endogenous Ras-GTP. B-D, Transfection of a constitutively active Ras mutant (Rasv12) sensitizes TRAIL-resistant OSCC3 cells to TRAIL-induced apoptosis (B) and cleavage of caspase 8, 3 and PARP. *Lower blot* shows the expression of Ha-RasV12 mutant in the transfected cells. (C). Cells were transiently transfected with an empty pcDNA3 vector or pcDNA3-RasV12 plasmid at a transfection efficiency of approximately 70%. After 24 h post-transfection, cells were treated with TRAIL at the indicated doses, and analyzed by flow cytometry as in 1B, and by immunoblotting for caspase-8, caspase-3 and PARP.

We asked if the introduction of Ras could convert an insensitive OSCC line to a sensitive one. To this end, OSCC3 cells were transfected with an empty vector or vector expressing a constitutively active H-Ras mutant (H-RasV12). Compared to empty vector, transfection of H-RasV12 significantly sensitized OSCC3 cells to apoptosis by rhTRAIL or anti-DR5, but not anti-DR4 antibody (Fig. 5B). In consistent, TRAIL induced an increase in cleavage of caspase-8 and caspase-3 in a dose- and time- dependent manner in H-RasV12 transfected cells (Figs. 5C & D). Thus, our results indicate that Ras activity plays a role in determining the cellular sensitivity to rhTRAIL or monoclonal antibody against DR5.

### Ras regulates the surface expression of DR5

**Figure 6:**
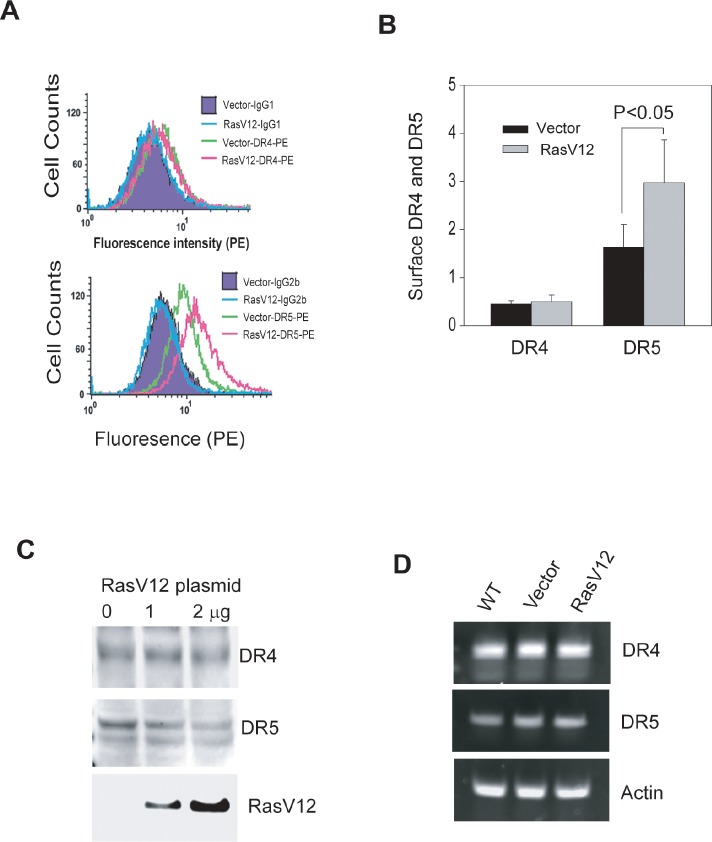
Ras activation upregulates DR5 expression on cell surface A & B, Transfection of dominant active RasV12 mutant in OSCC3 cells increases surface expression of DR5, but not DR4. Transfections were performed same as in 4B, and the resultant cells were analyzed by flow cytometry after labeling with PE-conjugated anti-DR4 or anti-DR5 as described in 3A. The quantification results (B) highlight a significant increase in DR5 surface expression upon RasV12 expression. C & D, Transfection of OSCC3 cells with RasV12 plasmid (1 or 2 μg) does not affect total protein and mRNA expressions of both DR4 and DR5. For detecting total proteins (C), equal amounts of whole cell lysates from the indicated cell lines were analyzed by immunoblotting using antibodies against DR4, DR5 and HA, respectively. For mRNA expression (D), extracted total mRNA samples were subjected to RT-PCR analyses using primers specific to DR4 or DR5 with β-actin being as an internal control.

To extend our finding beyond the association between Ras activity and TRAIL sensitivity, we addressed the issue of whether Ras regulates the expression of TRAIL death receptors. By flow cytometry, we found that the surface expression of DR5 was significantly upregulated in OSCC3 cells after transfection of RasV12 plasmid (Fig. 6A & B). However, expression of RasV12 had little effect on DR4 surface expression. Further, there was no change in the total mRNA and protein expression of both receptors (Figs. 6C & D). These results demonstrate that Ras is selectively involved in regulation of the expression of TRAIL receptor DR5 on cell surface.

## DISCUSSION

The development of molecularly targeted therapies holds promise in improving the outcome of metastatic oral squamous cell carcinoma (OSCC) – a disease with consistently poor prognosis for decades. Recent advance in understanding the molecular mechanisms of the disease has suggested several therapeutic targets such as signaling components of EGFR-Ras and PI3K-AKT-mTOR pathways [2]. Our study demonstrates that TRAIL receptor targeted agents may have therapeutic potential against OSCC. Both rhTRAIL and its agonistic antibodies induced rapid apoptosis in OSCC cell lines, especially in the ones harboring high Ras activity. The anticancer activity of rhTRAIL was confirmed in a relevant animal model. We also provide evidence that Ras is involved in regulation of surface expression of DR5. Ras is frequently hyperactivated in OSCC tumors [2, 27-29], thus TRAIL receptor targeted agents could be evaluated for use in the treatment of this malignancy.

The selectivity of TRAIL towards cancer cells has stimulated clinical investigations of agents targeting the TRAIL death receptors, including recombinant human TRAIL and monoclonal antibodies against DR4 and DR5. Early clinical trial data showed a general safety of these agents [15]. In anticipate tumor resistance, however, a challenge remains to identify the appropriate patient population that could be beneficial from the treatments with these agents. This requires identification of biomarkers that can predict tumor resistance to these agents. We have previously shown that TRAIL death receptors, with a higher incident in DR4, undergo constitutive endocytosis in breast cancer cells that results in their deficiency on cell surface and cellular resistance to the targeted therapies [21-23]. Our recent work made a similar observation in rhabdomyosarcoma cells [19]. In this study we found that DR4, but not DR5, is deficient on the surface of HN6 OSCC cell line despite its total protein expression (Fig. 4A). Lack of surface DR4 rendered the cells completely resistant to anti-DR4 antibody (Fig. 2C). Collectively, these data are supportive of our notion that surface deficiency of DR4 and/or DR5 could be used as a biomarker to exclude patients from TRAIL receptor targeted therapies.

As with many other cancer types, the development of oral cancer is often accompanied by an upregulated Ras signaling pathway due to gain of function mutations in *ras* genes itself and/or alterations in the regulatory proteins [2, 27-29]. The hyperactivity of Ras pathway is linked to tumor resistance to the approved EGFR antibody therapy [40, 41]. In contrast to its best known function in prompting cell growth, Ras has also been implicated in activation of cell death pathways [42]. Our data reveal a close link between Ras activity (Ras-GTP level) and TRAIL sensitivity in OSCC cell lines. Among the five cell lines tested, Ras-GTP levels were strongly associated with cellular sensitivities to rhTRAIL and the agonistic antibodies. Transfection of OSCC3 cells with H-RasV12, a constitutively active H-Ras mutant, increased apoptotic responses to rhTRAIL and anti-DR5 antibody (Fig. 5B). Thus, TRAIL based therapies may be useful in treating tumors that contain Ras mutations. In this context, Ras mutations have been shown to upregulate the total protein expressions of both DR4 and DR5 in colon cancer cell lines [24, 39]. Strikingly, expression of RasV12 selectively upregulated surface expression of DR5, but not DR4, while it had little or no effect on their total mRNA and protein expressions (Fig. 6). To our knowledge, this is the first evidence that Ras is directly involved in regulation of surface expression of TRAIL death receptors. Although the underling mechanisms remain to be elucidated, Ras has been implicated in the trafficking and endocytosis signaling of many other receptors. We are currently in the process to identify the signaling components that medicate Ras regulated upregulation of DR5.

In summary, we demonstrate a high degree of OSCC sensitivity to TRAIL receptor targeted agents. We also found a correlation between Ras activity and TRAIL sensitivity in OSCC cell lines. These data warrant additional studies to evaluate the potential use of rhTRAIL and its agonistic antibodies in treating these malignancies.

## MATERIALS AND METHODS

### Cell lines and reagents

The human head and neck cancer cell lines HN31, HN30, HN4, HN6, HN12, Cal27 and OSCC3 were from Dr. Silvio Gutkind at the National Institutes of Health, Bethesda. All cell lines were maintained in DMEM supplemented with 10% fetal calf serum, penicillin, and streptomycin. Recombinant human TRAIL (rhTRAIL), monoclonal antibodies specific to TRAIL death receptor-4 (AF347) and -5 (MAB6312) and their phycoerythrin (PE)-conjugated forms were purchased from R&D Systems (Minneapolis, MN). PE-conjugated antibodies to decoy receptor-1 (DcR1) and -2 (DcR2) were from eBioscience (San Diego, CA). The expression plasmid for a dominant active Ha-Ras mutant (RasV12) was obtained from University of Missouri-Rolla cDNA resource center. Transfections were performed using Neon electroporation transfection system (Invitrogen) per manufacturer's instruction.

### Cell viability assay

*C*ell viability was determined using a 3-(4,5-dimethylthiazol-2-yl)-2,5-diphenyltetrazolium bromide (MTT) colorimetric assay as previously described [36]. All experiments were performed in triplicate for each result.

### Apoptosis assay

Apoptosis was determined by flow cytometry as previously described [21]. Briefly, cells were grown on 6-well plates to ~80% confluence and treated with rhTRAIL or anti-DR4 or anti-DR5 antibodies at the indicated concentrations. At the indicated times, cells were harvested, stained with FITC-conjugated Annexin V and propidium iodide (PI), and analyzed by FACS Calibur and CellQuest software (Becton Dickinson).

### Flow cytometric analysis of surface DR4 and DR5 expression

Surface expression of DR4 and DR5 was determined by FACS analysis using PE-conjugated antibodies specific to the individual receptor and previously described [21]. In brief, cells were blocked with 1% goat serum, incubated with 10 μg/mL anti-DR4-PE or anti-DR5-PE (mouse IgG1-PE and IgG2b-PE as respective controls), and analyzed by flow cytometry.

### Immunoblot analysis

Cell lysates were prepared as previously described [37]. Protein concentrations were estimated using the BCA protein assay (Pierce, Rockford, IL). Equal amounts of cell lysates (20 μg/lane) were resolved by electrophoresis using a 4% to 12% NuPAGE Bis-Tris gel (Invitrogen) and transferred to PVDF membranes (Millipore) for immunoblot analysis. When necessary, the membranes were stripped by Restore Western Blot Stripping Buffer (Pierce) and reprobed with appropriate antibodies. Antibodies specific to human DR4, DR5, DcR1, and DcR2 were from Imgenex (San Diego, CA), antibodies to Bax and Bad were from BD Pharmingen (San Diego, CA), and antibodies to FLIP, PARP, caspase-3, caspase-8, XIAP-1, c-IAP-1 were from Cell Signaling Technology (Danvers, MA). Anti-actin was from Santa Cruz Biotechnology Inc. (Santa Cruz, CA).

### Ras activity assay

The levels of active GTP-Ras were determined by a pull-down assay using the Ras-interactive binding domain of human c-Raf-1 fused to glutathione-*S*-transferase (GST) that was immobilized on glutathione–Sepharose beads (GE Healthcare, Piscataway, NJ). Briefly, cells were grown in complete medium to 70–80% confluency, harvested, and lysed in a buffer containing 10 mM Tris, 100 mM NaCl, 1% Triton X-100, 0.5 mM EDTA, 40 mM β-glycerophosphate, 10 mM MgCl_2_, and protease inhibitors. Equal amounts (1.0 mg total proteins) of cell lysates were then incubated at 4°C for 30 min with glutathione–Sepharose beads containing 40 μg of GST-cRaf1. Ras-GTP bund to the beads pellets were then detected by immunoblotting using anti-Ras antibody.

### Reverse Transcription (RT) PCR

RT-PCR was carried out using High-Fidelity One-Step RT-PCR kit (Invitrogen) per manufacturer's instructions. Total cellular RNA was isolated using Trizol reagent (Invitrogen). Primer pairs used for detection of human DR4 and DR5 are as follows: DR4 (5-ATGGCGCCACCACCAGCTA and 5-CATGGGAGGCAAGCAAACA), and DR5 (5-AAGACCCTTGTGCTCGTTGTC and 5-GACACATTCGATGTCACTCCA). β-actin was used as an internal control. The RT-PCR products were resolved and visualized on 1% agarose gel.

### Orthotopic tumor xenografts in SCID/NOD mice

Animal studies were carried out as previously described [38] and followed the NIH-approved protocols (ASP#10-569) in compliance with the NIH Guidance for the Care and Use of Laboratory Animals. Female severe immunodeficient (SCID)/NOD mice (NCI), 4 to 6 weeks of age and weighing 18 to 20 g, were housed in appropriate sterile filter-capped cages, and fed and watered *ad libitum*. HN31 cells (1 × 10^5^) were injected directly into the dorsal tongue and the size (length and width) of lesions in the tongue was monitored weekly. At five weeks, total 8 animals with similar tumor volumes (~80 mm^3^) were randomly divided into two groups (4 animals per group), followed by intratumoral injections of 50 μl of PBS (Control group) or 50 μl of TRAIL (100 μg/ml). After 6 or 24 h post-injection, animals were euthanized and the tongues were dissected and processed for paraffin sectioning.

### Statistical analysis

Statistical analyses were performed with Sigma plot (Systat Software Inc., San Jose, CA). All cell-based studies were done in triplicate. Data were presented as mean + SD. Statistical comparisons were determined by Student's t-test. Statistical significance was defined as *P* < 0.05.

## References

[1] Conway DI, Hashibe M, Boffetta P, Wunsch-Filho V, Muscat J, La VC (2009). Enhancing epidemiologic research on head and neck cancer: INHA. Oral Oncol.

[2] Molinolo AA, Amornphimoltham P, Squarize CH, Castilho RM, Patel V, Gutkind JS (2009). Dysregulated molecular networks in head and neck carcinogenesis. Oral Oncol.

[3] Kreppel M, Drebber U, Eich HT, Dreiseidler T, Zoller JE, Muller RP (2011). Combined-Modality Treatment in Advanced Oral Squamous Cell Carcinoma : Primary Surgery Followed by Adjuvant Concomitant Radiochemotherapy. Strahlenther Onkol.

[4] Choong N, Vokes E (2008). Expanding role of the medical oncologist in the management of head and neck cancer. CA Cancer J Clin.

[5] Jerjes W, Upile T, Petrie A, Riskalla A, Hamdoon Z, Vourvachis M (2010). Clinicopathological parameters, recurrence, locoregional and distant metastasis in 115 T1-T2 oral squamous cell carcinoma patients. Head Neck Oncol.

[6] Ashkenazi A, Dixit VM (1998). Death receptors: signaling and modulation. Science.

[7] Sprick MR, Weigand MA, Rieser E, Rauch CT, Juo P, Blenis J (2000). FADD/MORT1 and caspase-8 are recruited to TRAIL receptors 1 and 2 and are essential for apoptosis mediated by TRAIL receptor 2. Immunity.

[8] Pollack IF, Erff M, Ashkenazi A (2001). Direct stimulation of apoptotic signaling by soluble Apo2l/tumor necrosis factor-related apoptosis-inducing ligand leads to selective killing of glioma cells. Clin Cancer Res.

[9] Gazitt Y (1999). TRAIL is a potent inducer of apoptosis in myeloma cells derived from multiple myeloma patients and is not cytotoxic to hematopoietic stem cells. Leukemia.

[10] Ashkenazi A (2002). Targeting death and decoy receptors of the tumour-necrosis factor superfamily. Nat Rev Cancer.

[11] Ashkenazi A, Pai RC, Fong S, Leung S, Lawrence DA, Marsters SA (1999). Safety and antitumor activity of recombinant soluble Apo2 ligand. J Clin Invest.

[12] Daniel D, Yang B, Lawrence DA, Totpal K, Balter I, Lee WP (2007). Cooperation of the proapoptotic receptor agonist rhApo2L/TRAIL with the CD20 antibody rituximab against non-Hodgkin lymphoma xenografts. Blood.

[13] Kelley SK, Harris LA, Xie D, Deforge L, Totpal K, Bussiere J (2001). Preclinical studies to predict the disposition of Apo2L/tumor necrosis factor-related apoptosis-inducing ligand in humans: characterization of in vivo efficacy, pharmacokinetics, and safety. J Pharmacol Exp Ther.

[14] Jin H, Yang R, Fong S, Totpal K, Lawrence D, Zheng Z (2004). Apo2 ligand/tumor necrosis factor-related apoptosis-inducing ligand cooperates with chemotherapy to inhibit orthotopic lung tumor growth and improve survival. Cancer Res.

[15] Ashkenazi A, Holland P, Eckhardt SG (2008). Ligand-based targeting of apoptosis in cancer: the potential of recombinant human apoptosis ligand 2/Tumor necrosis factor-related apoptosis-inducing ligand (rhApo2L/TRAIL). J Clin Oncol.

[16] Johnstone RW, Frew AJ, Smyth MJ (2008). The TRAIL apoptotic pathway in cancer onset, progression and therapy. Nat Rev Cancer.

[17] Irmler M, Thome M, Hahne M, Schneider P, Hofmann K, Steiner V (1997). Inhibition of death receptor signals by cellular FLIP. Nature.

[18] Deveraux QL, Roy N, Stennicke HR, Van Arsdale T, Zhou Q, Srinivasula SM (1998). IAPs block apoptotic events induced by caspase-8 and cytochrome c by direct inhibition of distinct caspases. EMBO J.

[19] Kang Z, Chen J, Yu Y, Li B, Sun SY, Zhang B (2011). Drozitumab, a human antibody to death receptor 5, has potent anti-tumor activity against rhabdomyosarcoma with the expression of caspase-8 predictive of response. Clin Cancer Res.

[20] Wagner KW, Punnoose EA, Januario T, Lawrence DA, Pitti RM, Lancaster K (2007). Death-receptor O-glycosylation controls tumor-cell sensitivity to the proapoptotic ligand Apo2L/TRAIL. Nat Med.

[21] Zhang Y, Yoshida T, Zhang B (2008). TRAIL induces endocytosis of its death receptors in MDA-MB-231 breast cancer cells. Cancer Biol Ther.

[22] Zhang Y, Zhang B (2008). TRAIL resistance of breast cancer cells is associated with constitutive endocytosis of death receptors 4 and 5. Mol Cancer Res.

[23] Yoshida T, Zhang Y, Rivera Rosado LA, Zhang B (2009). Repeated treatment with subtoxic doses of TRAIL induces resistance to apoptosis through its death receptors in MDA-MB-231 breast cancer cells. Mol Cancer Res.

[24] Drosopoulos KG, Roberts ML, Cermak L, Sasazuki T, Shirasawa S, Andera L (2005). Transformation by oncogenic RAS sensitizes human colon cells to TRAIL-induced apoptosis by up-regulating death receptor 4 and death receptor 5 through a MEK-dependent pathway. J Biol Chem.

[25] Nesterov A, Nikrad M, Johnson T, Kraft AS (2004). Oncogenic Ras sensitizes normal human cells to tumor necrosis factor-alpha-related apoptosis-inducing ligand-induced apoptosis. Cancer Res.

[26] Wang Y, Engels IH, Knee DA, Nasoff M, Deveraux QL, Quon KC (2004). Synthetic lethal targeting of MYC by activation of the DR5 death receptor pathway. Cancer Cell.

[27] Kuo MY, Jeng JH, Chiang CP, Hahn LJ (1994). Mutations of Ki-ras oncogene codon 12 in betel quid chewing-related human oral squamous cell carcinoma in Taiwan. J Oral Pathol Med.

[28] Schubbert S, Shannon K, Bollag G (2007). Hyperactive Ras in developmental disorders and cancer. Nat Rev Cancer.

[29] Sogabe Y, Suzuki H, Toyota M, Ogi K, Imai T, Nojima M (2008). Epigenetic inactivation of SFRP genes in oral squamous cell carcinoma. Int J Oncol.

[30] Chin L, Tam A, Pomerantz J, Wong M, Holash J, Bardeesy N (1999). Essential role for oncogenic Ras in tumour maintenance. Nature.

[31] Li W, Zhu T, Guan KL (2004). Transformation potential of Ras isoforms correlates with activation of phosphatidylinositol 3-kinase but not ERK. J Biol Chem.

[32] Itashiki Y, Harada K, Ferdous T, Yoshida H (2007). Effects of tumor necrosis factor-related apoptosis-inducing ligand alone and in combination with fluoropyrimidine anticancer agent, S-1, on tumor growth of human oral squamous cell carcinoma xenografts in nude mice. Anticancer Res.

[33] Noutomi T, Itoh M, Toyota H, Takada E, Mizuguchi J (2009). Tumor necrosis factor-related apoptosis-inducing ligand induces apoptotic cell death through c-Jun NH2-terminal kinase activation in squamous cell carcinoma cells. Oncol Rep.

[34] Yoshiba S, Iwase M, Kurihara S, Uchida M, Kurihara Y, Watanabe H (2011). Proteasome inhibitor sensitizes oral squamous cell carcinoma cells to TRAIL-mediated apoptosis. Oncol Rep.

[35] Uchida M, Iwase M, Takaoka S, Yoshiba S, Kondo G, Watanabe H (2007). Enhanced susceptibility to tumor necrosis factor-related apoptosis-inducing ligand-mediated apoptosis in oral squamous cell carcinoma cells treated with phosphatidylinositol 3-kinase inhibitors. Int J Oncol.

[36] Zhang B, Zhang Y, Dagher MC, Shacter E (2005). Rho GDP dissociation inhibitor protects cancer cells against drug-induced apoptosis. Cancer Res.

[37] Zhang Y, Rosado LA, Moon SY, Zhang B (2009). Silencing of D4-GDI Inhibits Growth and Invasive Behavior in MDA-MB-231 Cells by Activation of Rac-dependent p38 and JNK Signaling. J Biol Chem.

[38] Patel V, Marsh CA, Dorsam RT, Mikelis CM, Masedunskas A, Amornphimoltham P (2011). Decreased lymphangiogenesis and lymph node metastasis by mTOR inhibition in head and neck cancer. Cancer Res.

[39] Oikonomou E, Kosmidou V, Katseli A, Kothonidis K, Mourtzoukou D, Kontogeorgos G (2009). TRAIL receptor upregulation and the implication of KRAS/BRAF mutations in human colon cancer tumors. Int J Cancer.

[40] Dempke WC, Heinemann V (2010). Ras mutational status is a biomarker for resistance to EGFR inhibitors in colorectal carcinoma. Anticancer Res.

[41] Roberts PJ, Stinchcombe TE, Der CJ, Socinski MA (2010). Personalized medicine in non-small-cell lung cancer: is KRAS a useful marker in selecting patients for epidermal growth factor receptor-targeted therapy?. J Clin Oncol.

[42] Overmeyer JH, Maltese WA (2011). Death pathways triggered by activated Ras in cancer cells. Front Biosci.

